# Gene Therapy: Routes of Inner Ear Delivery

**DOI:** 10.1097/AUD.0000000000001771

**Published:** 2026-10-13

**Authors:** Erdem Yildiz, Christoph Arnoldner

**Affiliations:** 1 Christian Doppler Laboratory for Inner Ear Research, Division of Head and Neck Surgery, Department of Otorhinolaryngology, Medical University of Vienna, Vienna, Austria

**Keywords:** Cochlea, Delivery, Gene therapy, Inner ear, Local therapeutics, Viral vector

## Abstract

Gene delivery and the various routes of administration used to deliver viral vectors constitute a rapidly evolving field, with recent success in restoring hearing in children with *OTOF* variants in ongoing clinical otoferlin trials. With the rise of gene therapy for major hereditary hearing disorders, both syndromic and nonsyndromic, there has been parallel progress in the development of new delivery methods. Advances in intratympanic injection of antibiotics and corticosteroids, along with nearly half a century of experience in cochlear implantation surgery, have enabled multiple local and systemic approaches to access the inner ear. Recent industry investment in gene therapy and delivery technologies has accelerated scientific progress, underscoring the need for continuous evaluation of the most suitable gene delivery strategies for different causes of hearing loss. Although most gene delivery in animal models has been performed via round window membrane injection, yielding promising results in cochlear hair cell transduction, potential drawbacks remain for clinical translation—particularly limited vector distribution, which may result in a basal-to-apical gradient. Venting procedures such as stapes footplate fenestration or canalostomy have been explored to mitigate these limitations, and recent intrathecal or even in utero injections have been investigated as alternative approaches. Hearing loss can be inherited in multiple ways, and patient treatment with gene delivery, or even the type of gene therapy itself, may vary with the recent success of CRISPR/Cas-based editing tools. Understanding the genetic etiology of inner ear disorders has facilitated the identification of therapeutic targets and innovative delivery approaches. This review summarizes the current understanding of delivery routes for gene therapy, highlighting their respective strengths and limitations as demonstrated in translational and clinical research to date. Furthermore, it examines advances in drug delivery and clinical applications in the context of emerging therapeutics, providing an integrated overview and perspective on the future direction of gene delivery strategies.

## INTRODUCTION

Hearing constitutes a fundamental sense that plays a pivotal role in communication and social interaction. The ability to hear shapes how we connect with others and interpret our environment; thus, restoring auditory function through inner ear-targeted gene and drug delivery holds transformative potential. Congenital and acquired hearing loss remain major health challenges, with genetic etiologies accounting for a significant proportion. One out of every 1000 children is born with severe-to-profound hearing loss, underlining its major health burden that needs to be urgently addressed (Lieu et al. 2020). The pioneering work of Clark, Hochmair, and Wilson in the 1970s led to the development of the cochlear implant—widely regarded as the most successful neuroprosthetic device to date. Until recently, it remained the only clinical option for restoring hearing in individuals with severe to profound sensorineural hearing loss. Despite this breakthrough, the limitations that go along with the implanted devices result in unsatisfactory outcomes in nearly one out of five individuals (Holden et al. 2013; Beyea et al. 2016). This has spurred interest in novel, biologically mediated therapy alternatives, particularly gene therapy, with a promising avenue for targeted intervention. The successful application of lipid nanoparticle–mediated gene editing in a rare metabolic disorder highlights the clinical viability of personalized therapies (Musunuru et al. 2025), offering a model that may be extended to hearing loss and related conditions.

The early 2000s can be described as a scientific stage for the development of gene-based interventions in translational hearing research. Since then, gene therapy has emerged as a promising approach for the cure of various types of hearing loss in preclinical animal studies. Advances in sequencing technologies, such as next-generation sequencing and whole-exome sequencing, have enabled the identification of many genes associated with hearing loss, offering precise molecular targets for interventions. Concurrently, the development of various viral vectors (Table 1), including adeno-associated viruses (AAVs), adenoviruses (AVs), and lentiviruses, has facilitated efficient gene delivery into target tissues, significantly advancing gene therapy explorations for the treatment of inner ear diseases. While gene therapy presents an exciting opportunity for hearing restoration, one of its greatest challenges lies in the efficient and safe delivery to the inner ear. The presence of the blood–labyrinth barrier restricts the passage of systemically administered therapeutics, reducing their bioavailability in cochlear sensory cells (Nyberg et al. 2019). This necessitates the development of precise and localized delivery methods.

**TABLE 1.** Comparison of currently used common formulations of drug or gene vehicles

Formulation	Size		Reference
Viral vectors	Adeno-associated virus Adenovirus Lentivirus Herpes simplex virus	20–30 nm 70–90 nm 80–120 nm ~150 nm	Drouin and Agbandje-McKenna (2013) Lopes da Silva (2011) Segura et al. (2013) Zhou et al. (2000)
Non-viral vectors	(Magnetic) Nanoparticles, liposomes, polymersomes	1–100 nm	Pyykko et al. (2016)
Gel formulations with nanoparticles	Microemulsion gel formulation of Triamcinolone-acetonide Polylactid-co-glycolid nanoparticle formulation with dexamethasone	16.5 nm 150 nm	Nguyen et al. (2023) Kim et al. (2021)

Currently, gene therapy approaches use primarily local delivery strategies. Intratympanic (IT) administration involves injecting the therapeutic agent into the middle ear, where it diffuses across barriers such as the round window membrane (RWM) and the oval window, ultimately reaching the perilymphatic space (Glueckert et al. 2018). This method helps minimize systemic side effects, but its efficacy is often hindered by variable diffusion rates, clearance via the eustachian tube, and inconsistent drug penetration across cochlear regions. To address these challenges, alternative strategies, such as the use of hydrogels, nanoparticles, and magnetically guided delivery systems, have been explored to enhance local retention and controlled release (Delaney et al. 2023). Intracochlear gene delivery, though more invasive, enables more precise control over vector distribution within the cochlea. Direct microinjection of viral vectors into the scala media or scala tympani ensures precise targeting of cochlear cells but carries the risk of inner ear trauma and potential hearing damage. A promising avenue to mitigate these risks is the integration of gene therapy delivery with cochlear implants. Studies have demonstrated that cochlear implant electrode arrays can serve as a conduit for delivering drugs directly into the inner ear, leveraging the proximity of the electrode to sensory structures while simultaneously attempting to restore auditory function (Arambula et al. 2022). This dual-function approach represents an innovative synergy between bioelectronic devices and potential gene delivery that is currently investigated in a clinical trial for cochlear implant-driven neurotrophins (St Peter et al. 2022; Birman).

Beyond gene therapy, drug delivery strategies have also evolved to address the challenges associated with treating inner ear disorders. Localized delivery methods, including sustained-release formulations (Honeder et al. 2019), nanoparticle-based carriers (Pyykko et al. 2016), and microfluidic pumps (Chen et al. 2005), are explored to enhance the therapeutic efficacy of active agents. The translation of gene therapy and advanced drug delivery methods into clinical practice remains a work in progress, requiring rigorous validation in preclinical models and human trials. Several hurdles must be overcome to increase the efficiency and safety in human trials, including optimizing viral vector efficiency, minimizing immune responses, and ensuring long-term safety.

## BASIC INNER EAR ANATOMY

Drug and gene delivery approaches are dependent on many different parameters such as size, polarity, and concentration of the active substance, and the success of any novel therapeutic depends on the pharmacokinetic profile of the individual agent and the correct assessment of the benefit-to-risk ratio. Fundamental knowledge of labyrinthine anatomy, auditory physiology, and the underlying pathomechanisms of genetic disorders is essential for designing gene delivery approaches to treat hereditary hearing loss. The outer ear collects sound waves, which travel into the external auditory canal toward the tympanic membrane, causing a vibration of the ossicles that amplifies the sound and transmits vibration energy to the oval window via the stapes footplate. Oval and round windows are two membranous structures of the labyrinth with slight mobility and lie directly adjacent to the intracochlear fluid space, offering unique opportunities for drug delivery to the inner ear. Without its dynamic and fluid-filled characteristics, cochlear mechanotransduction, the conversion of acoustic energy into electrical signals, would fail, resulting in hearing loss. The spiral-shaped cochlea contains three different fluid compartments: the *scala vestibuli* (vestibular duct), *scala media* (cochlear duct), and the *scala tympani* (tympanic duct). The basilar membrane separates the perilymphatic space of the *scala tympani* from the endolymphatic space of the *scala media*. The organ of Corti, which rests on the basilar membrane, contains outer hair cells with tip links on the stereocilia that induce ion channel openings and ultimately lead to the generation of action potentials. Equally essential for maintaining ion homeostasis—and of indirect but significant importance—is the blood–labyrinth barrier, whose tight junctions regulate the exchange of substances between the bloodstream and the inner ear fluid spaces (Nyberg et al. 2019). Considering the complexity of the auditory pathway, its many relay points in the transmission of sound (e.g., ossicles, basilar membrane function, organ of Corti) can be impaired at various levels, either through acquired or genetic hearing loss. The underlying pathomechanism of the hearing impairment needs to be clarified to spot the correct diagnosis and treatment. This is particularly important when selecting the most suitable genes for treatment and gene-delivery tools that efficiently target the affected cells while minimizing damage to the target organ or off-target tissues.

The research explorations of the past decade have been crucial in understanding an improved, safe, effective, and precise delivery. So far, the most promising results have been shown with viral vectors such as AAVs, AVs, and retroviruses, as they have a high transduction efficiency and can induce long-term, high expression of transgenes. Each of the mentioned viral vectors has its advantages and limitations, with ongoing research efforts aimed at overcoming these challenges. For example, the dual AAV vector approach utilizes AAV vectors in which the transgene is split into multiple segments that are subsequently reassembled within the target cell, enabling the delivery of larger genetic cargo (Duan et al. 2003). Another example is the helper-dependent adenovirus that contains only the transgene and essential regulatory elements, thereby reducing the immune response (Liu & Seol 2020; Ricobaraza et al. 2020).

## DELIVERY ROUTES: SYSTEMIC VERSUS LOCAL VIRAL VECTOR DELIVERY

Systemic drug delivery for the treatment of inner ear diseases has a long history in clinical medicine. Streptomycin, the first discovered antibiotic, was systemically administered to soldiers and revealed an off-target vestibulo-ototoxic effect in patients (Fowler & Seligman 1947). Later, this side effect was used for the treatment of Menière’s disease to ablate vestibular hair cells in patients, ultimately alleviating vertigo symptoms (Webster et al. 1970). Systemic delivery has its advantages; intravenous (IV), intramuscular (IM), subcutaneous (SC) injections along with oral drug intake, are relatively easy to perform, and physicians have usually the most experience using these administration methods (Fig. 1A). However, a classic example of many detrimental side-effects is the systemic use of corticosteroids to treat idiopathic sensorineural hearing loss in patients (Plontke et al. 2024). The inner ear is a well-isolated organ in the temporal bone, and the protective blood–labyrinth barrier not only keeps disturbances such as infections away, but also requires high doses of systemic administration to achieve the required concentration of the active ingredient within the inner ear (Bird et al. 2007). Nevertheless, IV AAV delivery has been shown to induce high transduction in inner hair cells, spiral ganglion neurons, and vestibular hair cells in mice (Shibata et al. 2017). However, high doses of systemically delivered viral vectors can trigger immune responses that lead to toxicity—for instance, elevated transaminase levels indicative of liver toxicity (Colella et al. 2018). Due to their specific tropism, some viral vectors can target multiple organs (D. Wang et al. 2019). Although the risk of host-genome integration is low, the potential for genotoxicity remains, particularly at high doses (Suoranta et al. 2022). To overcome these challenges, superparamagnetic iron oxide nanoparticles were coupled with various AAV serotypes and administered intravenously in rats. An external magnetic field (1 Tesla) was then applied near the left ear to guide vector accumulation and promote passage across the blood‑labyrinth barrier (Ferdous et al. 2025). This approach enabled targeted transduction of cochlear structures, including hair cells and spiral ganglion neurons. Nevertheless, multiple off‑target organs—including brain, heart, kidney, liver, and spleen—also exhibited vector uptake and BDNF expression, underscoring the need for further refinement of vector specificity and targeting efficiency.

**Fig. 1.**
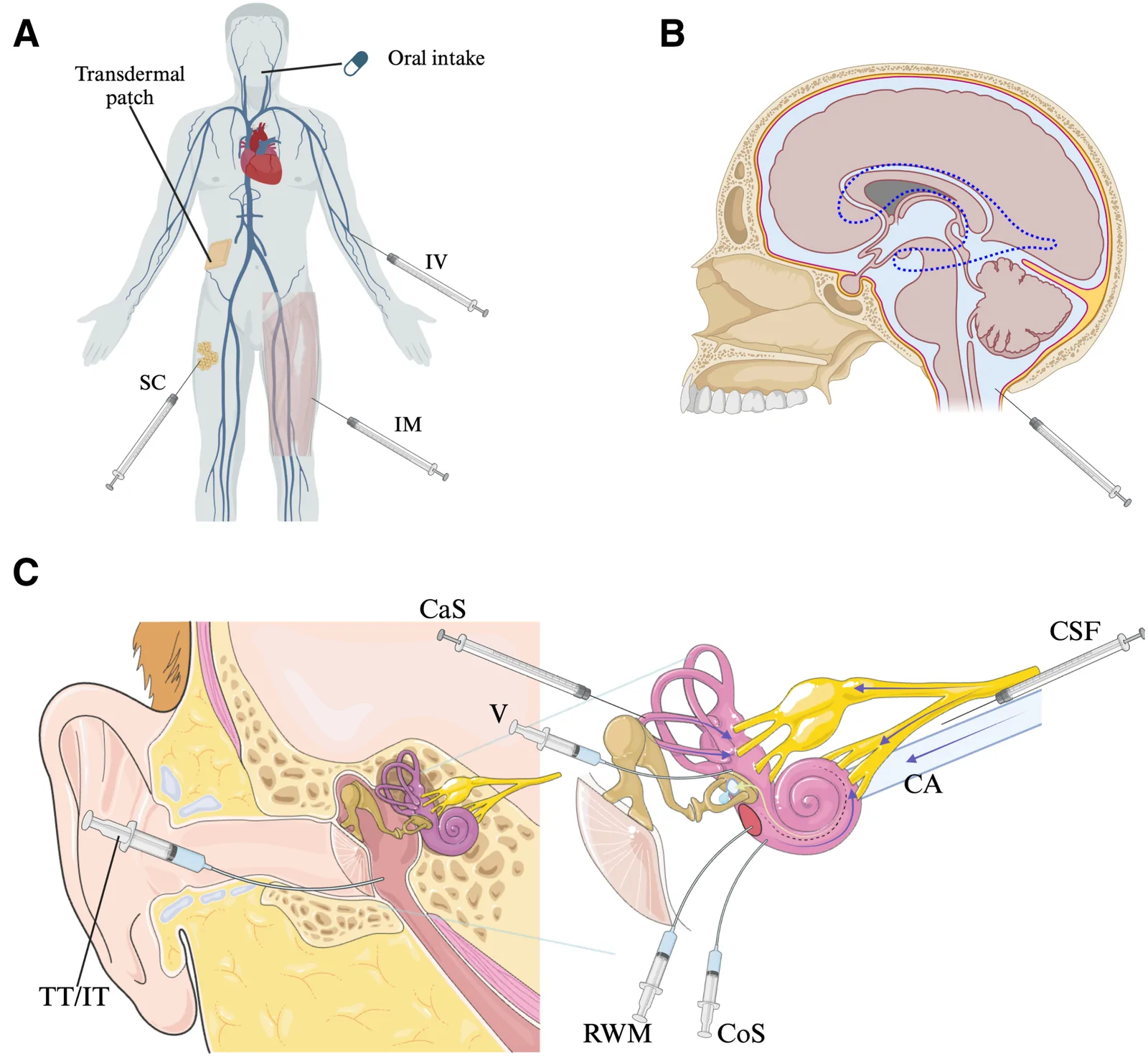
A, Systemic delivery routes with an illustration of the human body, including veins in blue, muscle, and fat tissue. Intravenous (IV), intramuscular (IM), subcutaneous (SC). B, Schematic illustration of a sagittal cut of the human head. An intrathecal injection route via the cerebellomedullary cistern into the cerebrospinal fluid is presented. C, Illustration of local delivery routes to the middle and inner ear space. Transtympanic (TT), intratympanic (IT), round window membrane (RWM, shown as a red circle), cochlear aqueduct (CA), cerebrospinal fluid (CSF), canalostomy (CaS), cochleostomy (CoS). The figure was created and adapted from the Servier Medical Art (https://smart.servier.com/; CC BY 4.0) in combination with Biorender.com.

As an alternative to systemic delivery, administration into the cerebrospinal fluid (CSF) space offers another route to the inner ear (Fig. 1B), as the cochlea is connected to the CSF via the cochlear aqueduct (Blanc et al. 2021; Mathiesen et al. 2023; Ranum et al. 2023). This route has primarily been used to treat neurological disorders (J. Hu et al. 2023), but has also been applied for CSF leak repair following intrathecal injection of sodium fluorescein (Kirchner & Proud 1960). The first intrathecal viral vector administrations demonstrated efficient inner ear transduction of AAVs. It is interesting that minimal off-target effects and no inflammatory responses have been reported (Blanc et al. 2021; Mathiesen et al. 2023). IT (Fig. 1C) drug delivery has its origins back in the 1870s, when Adam Politzer made several milestone contributions for the treatment of ear diseases. He introduced one of the first grommets that was inserted to the middle ear via myringotomy, a predecessor of today’s modern myringotomy tubes (Politzer 1981). However, it was only Harald Schuknecht in 1956 who did the first modern attempt of IT streptomycin drug delivery to treat patients with Menière’s by ablation of hair cells (Schuknecht 1956). Schuknecht conceived the idea of IT aminoglycoside injection as an adaptation of the established practice of IT formalin delivery, which was used to preserve inner ear tissue postmortem for histological analysis. His observations showed that both formalin and streptomycin seem to permeate the inner ear with varying times (Schuknecht 1957; Lustig 2004). Despite a few attempts, IT delivery remained unpopular until the 1990s.

Especially the inner ear seems to be well-suited for gene therapy strategies due to its isolated and protected location within the temporal bone. When combined with a safe surgical approach, this strategy minimizes off-target effects and subsequently elicits a low immune response, thereby reducing the risk of potential toxicity—particularly considering the protective role of the blood–labyrinth barrier. Local drug delivery strategies of small-molecule particles (non-viral) have shown various insights into inner ear pharmacokinetics in both small and large animal models (Mynatt et al. 2006; Plontke et al. 2007; Yildiz et al. 2023). The key advantage of the local delivery method is the decreased off-target effect in comparison to systemic administration, as it has been well-described in the treatment with high-dose corticosteroids (Plontke et al. 2024). Therefore, IT gene delivery routes using different viral vectors and concentrations are currently under investigation. For example, a minimally invasive surgical method to deliver AAV8-mut733 across the RWM involves gently placing gelfoam on the membrane before injecting 10 µL of the viral vector. This gelfoam-assisted delivery was used in transgenic mice to induce AAV-mediated overexpression of an X-linked apoptosis protein inhibitor before cisplatin treatment (Jie et al. 2015).

Other middle ear drug depot alternatives include biodegradable implants that can be placed in contact with the RWM to facilitate the sustained release of small molecules for inner ear disease treatment (Plontke et al. 2022). It is important to note, however, that gene therapy typically involves the use of viral vectors, which are substantially larger than conventional small-molecule drugs. For example, AAVs measure approximately 20 to 30 nm in diameter (Drouin & Agbandje-McKenna 2013), AVs 70 to 90 nm (Lopes da Silva 2011), lentiviruses 80 to 120 nm (Segura et al. 2013), and herpes simplex virus type 1 up to ~150 nm (Table 1), (Zhou et al. 2000). In contrast, commonly studied small-molecule drugs such as triamcinolone acetonide or dexamethasone are only ~1 to 2 nm in size. Nonetheless, larger-sized delivery systems—including special microemulsion gel formulations (~16.5 nm) and polylactide-co-glycolide nanoparticles (~150 nm)—have also been successfully applied to the inner ear (Kulkarni & Shaw 2016; D. H. Kim et al. 2021). These size differences may significantly influence pharmacokinetic behavior, particularly in the context of local gene delivery. To address this size issue, IT delivery using various gene carriers is currently under investigation. For example, extracellular vesicles derived from porcine RWM have been proposed as novel vehicles for gene and drug delivery. These vesicles exhibit natural membrane compatibility and may enable atraumatic passage across the RWM, thereby facilitating the effective delivery of therapeutic cargo—including AAV vectors and neurotrophic factors—across biological barriers (Holdsclaw et al. 2025). Another promising approach for delivering larger genetic payloads involves polyamidoamine dendrimers, which demonstrate stable DNA complexation and promote cellular uptake and gene transfection. When combined with a thermosensitive hydrogel based on hexanoyl glycol chitosan, they form an injectable IT matrix with the potential for sustained gene release into the inner ear. This system has shown enhanced gene expression in cochlear tissues (Le et al. 2025).

## DIRECT INTRACOCHLEAR DELIVERY

Intracochlear vector delivery requires extensive anatomical knowledge of the temporal bone in the treated species. The most common approach for gene delivery has been the intracochlear injection via the RWM (Table 2). Another common route involves the opening of one semicircular canal, also referred to as the canalostomy (CaS). Whereas the posterior semicircular canal (SCC) can be more easily accessed in mice, the lateral (horizontal) SCC offers easier access in humans. Vector delivery via the posterior SCC with the CaS approach was efficient in transduction using AAVs in mice cochleae (Suzuki et al. 2017), and CaS approach for vector delivery in larger animals has not been commonly used. Cochleostomy (CoS) refers to the drilling of the bony wall of the cochlea, usually facilitating cochlear implant surgery (Richard et al. 2012). Viral vector injection through the RWM appears to be the most logical and direct approach, and has been performed primarily in rodents (Bankoti et al. 2021), mostly in newborn mice before hearing onset, which occurs around 2 weeks after birth in mice (Mikaelian & Ruben 1965). In contrast, auditory function in humans is documented to begin around gestational week 19 (Hepper & Shahidullah 1994). Gene therapy has only recently been successful via the RWM approach in adult mice (Akil et al. 2019; Du et al. 2023; Tang et al. 2023), and the intracochlear delivery method has been predominantly used as it allows high concentration of therapeutic vectors with potential low immunogenic responses in off-target tissue. However, the structural layers of the RWM, including its associated blood vessels, may be compromised during puncture, potentially allowing unintended leakage of gene vectors into the systemic circulation and resulting in off-target transduction in other organs. In most recent studies with nonhuman primates and in clinical trials, the RWM approach has been adopted and revealed almost no injection-associated damage (Lv et al. 2024; Qi et al. 2024a). Despite opening the inner ear to administer a viral vector through the RWM, the risk of structural damage to the organ of Corti, and subsequently leading to functional decline, appears to be low. The viral suspension may come in contact with the basilar membrane, potentially accessing the endolymphatic space to transduce in targeted cells. However, this technique most likely depends on the proficiency of the surgeon and the use of an advanced injection system such as a micropump. In humans and nonhuman primates, otologists have the most experience with this approach, as traditional cochlear implantation has been performed via a mastoidectomy and posterior tympanotomy to access the RWM. A transcanal endoscopic approach with microinjection through the RWM has recently been proposed for AAV delivery in patients, based on cadaveric testing (L. Zhang et al. 2025). In addition, it is a common strategy to plug the RWM perforation after vector delivery using autologous fascia tissue to prevent perilymph leakage (Yoshimura et al. 2018).

**TABLE 2.** Advantages and disadvantages of common delivery routes of gene therapy

Delivery Method	Advantage	Disadvantage
Round window membrane	• Established procedure • Opening through easy puncture of the RWM • Safe procedure with low risk	• Potential loss of viral vectors via the cochlear aqueduct • Potential elevation of hearing thresholds
Canalostomy	• Safe procedure with low risk • Potentially higher efficiency to target the vestibular system	• Can cause vestibular deficits • Potential fibrosis of the semicircular canal can lead to vestibular disorders • Limitation of delivery speed and volume • Less established process
Cochleostomy	• Established surgical approach	• Potential damage to the inner ear structures may lead to irreversible hearing loss • Disturbance of ionic homeostasis • Potential loss of active agent via the cochlear aqueduct
Cerebrospinal fluid	• No transmatoid surgery necessary due to direct intrathecal injection	• Potential transduction and immunogenic response in the central nervous system
Systemic (IV, IM, SC, Transdermal, intake)	• Easy handling	• Immunogenic response and off-target effects of viral vector delivery

IV, intravenous; IM, intramuscular; RWM, round window membrane; SC, subcutaneous.

However, the overpressure of perilymph during RWM injection may result in the loss of viral vectors via the cochlear aqueduct (Talaei et al. 2019). This can vary in humans in terms of patency, depending primarily on patient age (Włodyka 1978; Bachor et al. 1997). Therefore, a fenestration (e.g., via a stapes venting hole as illustrated in Fig. 1C or a CaS before RWM injection) before injection of the viral vector through the RWM has been proposed to obtain a homogenous distribution from base to apex within the cochlea (Yoshimura et al. 2018; Andres-Mateos et al. 2022). This fenestration may prevent pressure buildup by releasing perilymphatic fluid, thereby reducing intracochlear trauma and improving the distribution of applied vectors. However, size and method of fenestration seem to be relevant; a stapes luxation may lead to a larger trauma and perilymph leakage compared with a CaS, as stapes luxation and its reattachment after model drug injection have been shown to lower drug concentrations when compared with direct intracochlear injection within the cochlea in a large animal model (Yildiz et al. 2023). The CaS approach implies the lateral SCC injection in humans, as it is the fastest accessible canal during traditional mastoidectomy surgery and is the shortest among all three SCCs, but the posterior and superior SCCs are also accessible for CaS in humans. Logically, this approach would enhance the transduction of the transgene of applied viruses in the vestibular system. Nonetheless, CaS opening may lead to potential disturbance of vestibular function when a large volume of fluid is delivered at high speed (Zhao et al. 2022). Some preclinical studies utilized adhesive materials (e.g., cyanoacrylate glue) to seal the injection site (J. Kim et al. 2024). It remains questionable whether this could be used reproducibly in humans, as one of the clinical *OTOF* trials has already reported potential leakage of the delivered viral vector from the RWM (Lv et al. 2024). In contrast to CaS, CoS involves drilling the wall at the promontory, potentially risking structural damage to the cochlea. With this method, a direct injection into the endolymphatic fluid (Fig. 1C) is feasible (Kilpatrick et al. 2011; Chien et al. 2015; Shu et al. 2016), but changes of the ion homeostasis and the higher trauma due to bone removal may lead to a higher risk of irreversible hearing loss. Alternative RWM injection methods have been proposed with the insertion of an inner ear catheter (Prenzler et al. 2018), and novel manufactured microneedle injection systems with the intention to minimize damage during delivery of the active agent (Szeto et al. 2020; Yu et al. 2020). Further refinements of those specialized delivery tools have enabled the development of ultra-sharp hollow microneedles, allowing for atraumatic access to the perilymph—either for precise injection or fluid sampling (Szeto et al. 2021). Recent advances include the design and in vivo testing of dual‑lumen microneedles capable of penetrating the RWM to deliver recombinant AAV vectors while simultaneously aspirating perilymph to maintain intracochlear pressure. In guinea pigs, this novel device facilitated delivery of 5 µL (around 50% of the total cochlear perilymph volume) of AAV‑GFP across the RWM with minimal hearing threshold shifts and achieved robust cochlear hair cell transduction following a characteristic base-to-apex gradient (Hammer et al. 2025). Although various delivery methods are under investigation, no standardized guideline has yet been established for selecting the most appropriate approach or the instruments used for drug delivery. Especially regarding injection speed during surgery and the resulting intracochlear pressure changes, evidence is limited. Moreover, procedures commonly used in translational experiments—for example, with devices connected via a micromanipulator or an injection pump in the microliter range—are often not translatable into clinical practice. However, three published clinical studies on *OTOF* gene therapy to date have used a similar setup for vector delivery. These systems typically consist of a syringe pump connected to a syringe, which is linked to an insertion device such as a catheter or needle (Fig. 2). Despite this convergence in hardware design, determining the optimal injection speed and volume for AAV administration remains challenging. For example, Regeneron’s recently published data demonstrated promising outcomes using a 240 µL vector administration designed to fully perfuse the cochlear perilymphatic space (Valayannopoulos et al. 2025), whereas other studies have reported comparable therapeutic effects with injection volumes ranging from 30 to 50 µL (H. Wang et al. 2024; Qi et al. 2025).

**Fig. 2.**
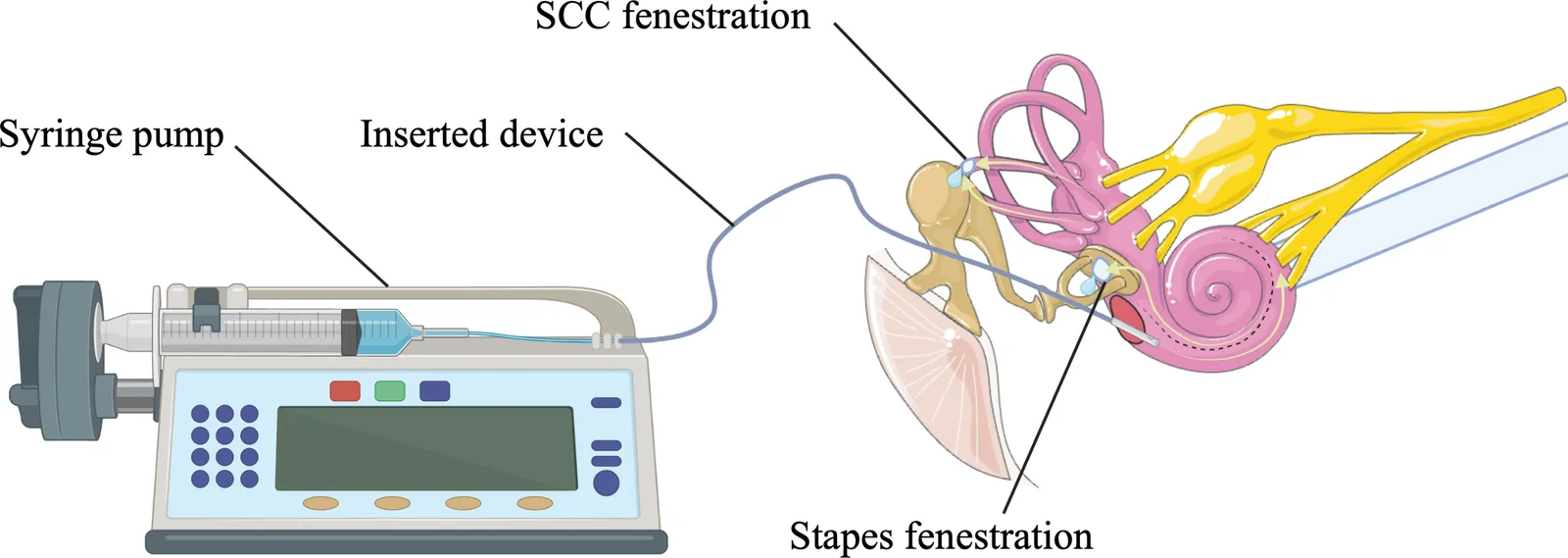
Schematic of a commonly used setup for atraumatic vector delivery into the inner ear. The system consists of a syringe pump connected to a syringe and an injection device (e.g., a catheter). Two fenestration sites are illustrated, representing potential openings—such as in the semicircular canal (SCC) or at the stapes footplate—that allow perilymph egress and relieve pressure resulting from the injected volume. The figure was created with Biorender.com.

## FUTURE DIRECTIONS AND CONCLUSION

Among the proposed local delivery routes, the ideal approach for gene therapy has not been determined. RWM injection has shown promising results in the transduction efficacy in translational experiments, but recent intrathecal injections indicate high intracochlear transduction rates after AAV injection via cerebellomedullary cistern in nonhuman primates (Ranum et al. 2023). In utero injections in mice have also been described with an AAV2/Anc80L65 vector and assessed high transduction efficacy in the inner ears (C. J. Hu et al. 2020). This embryonic delivery may be further investigated for the treatment of prenatal inner ear disorders, although clinical application may be challenging due to ethical considerations. All ongoing clinical trials to treat the autosomal recessive nonsyndromic deafness 9 (DFNB9 or *OTOF* gene) in pediatric patients rely on intracochlear injections, some including a fenestration method before injection. Two companies even developed a specialized device for vector delivery, which had to be tested for safety before use in the clinical routine (Table 3). Some delivery systems, such as inner ear catheters, are available for clinical use. Intracochlear delivery remains undoubtedly one of the major routes in future studies. Clearly, approaches that cause less damage to inner ear structures are favored. IT gene delivery may emerge as a viable administration method in the future, as advances in viral vector manufacturing are expected to reduce production costs, thereby permitting larger injection volumes into the middle ear space. Extended contact time between the injected solution and the RWM—before clearance via the Eustachian tube—may, in turn, enhance vector diffusion into the inner ear by sustaining the concentration gradient across the membrane (Patel et al. 2019). In addition, technological advancements may enhance efficiency while minimizing off-target effects, such as immunogenicity. Nevertheless, systemic administration remains a desirable option due to its major advantage of ease of delivery in humans. A recent clinical trial in patients with Menière’s disease treated with the orally administered small-molecule SPI-1005 (NCT04677972) further highlights the feasibility of non-invasive inner ear targeting (Kil 2024). In addition, the FDA approval of sodium thiosulfate (Pedmark, Fennec Pharmaceuticals Inc) IV use in children to prevent cisplatin-induced hearing loss demonstrates the clinical viability of systemic approaches to protect auditory function (Brock et al. 2018). Although not a gene therapy, these studies underscore the potential of systemic delivery routes, which may be explored for gene-based approaches, including oral administration of viral vectors in preclinical models, as it has been demonstrated that orally delivered AAV vectors can achieve transgene expression in intestinal tissues and correct metabolic dysfunction in a rodent model of lactose intolerance (During et al. 1998). This raises the question of whether a prolonged systemic delivery approach, such as via loaded dermal patches or similar alternatives, could be beneficial. There is no doubt that the clinical *OTOF* studies are already a major milestone in the treatment of prelingual hearing loss. However, the long-term outcomes following viral vector delivery in affected patients remain to be seen. Future clinical trials need to address questions of viral vector choice, volume, injection speed, and delivery route, leading to an optimized gene therapy. Companies such as Regeneron Pharmaceuticals (New York, NY) announced in their press release their clinical efforts with the AAV.103 vector for patients with GJB2-related hearing loss and the AAV.104 vector for people with stereocilin (STRC)-related hearing loss (Regeneron Pharmaceuticals 2024). In preclinical experiments of some research groups, GJB2 delivery has successfully restored auditory function (Ivanchenko et al. 2024). The continuously developing CRISPR/Cas-based editing tools (Clarissa et al. 2025) have also demonstrated efficient results in treating hereditary hearing loss after intracochlear delivery, providing tools to extend a wide range of applications that may enable gene expression specifically in cochlear cells. Clearly, the optimal gene therapy approach may be determined by assessing the highest possible transduction rate in the targeted sensory cells while minimizing injection-associated damage.

**TABLE 3.** Preclinical studies and clinical trials with local viral vector delivery into the inner ear of large species (nonhuman primates, NHPs, and humans)

Delivery Method	Viral Vector	Injected Species	Reference
RWM injection (facial recess)	AAV9-PHP.B	NHP (*Macaca fasciculari*s)	György et al. (2019)
RWM injection (facial recess)	AAV9-PHP.B	NHP (*Macaca fascicularis*)	Ivanchenko et al. (2020)
RWM injection (facial recess)	AAV-S	NHP (*Macaca fascicularis*)	Ivanchenko et al. (2021)
RWM injection with and without stapes fenestration and facial recess	Anc80L65 or AAV1	NHP (*Macaca mulatta*)	Andres-Mateos et al. (2022)
RWM injection with stapes fenestration and facial recess	AAV1-hOTOF	NHP (*Macaca fascicularis*)	L. Zhang et al. (2023)
RWM injection (facial recess)	AAV1 and AAV2/Anc80L65	NHP (*Macaca fascicularis*)	Qi et al. (2024b)
RWM injection (facial recess)	AAV-GRE-hsGJB2.HA	NHP (*Macaca fascicularis*)	Ivanchenko et al. (2024)
RWM injection with stapes fenestration 120 nL/sec injection speed	AAV1-hOTOF 1.5 × 10¹² vg Volume: 50 µL	Human	Refreshgene Therapeutics (Lv et al. 2024; Wang et al. 2024)
RWM injection with fenestration of the lateral SCC and facial recess DB-OTO delivery system using a Vygon Premicath catheter connected to a syringe and controlled by a syringe pump	DB-OTO (AAV1-hOTOFv5) 7.2 × 10^12^ vg/ear Volume: 240 µL	Human	Regeneron Pharmaceuticals (2024) and Valayannopoulos et al. (2025)
RWM injection with the Akouos delivery device and stapes fenestration and transcanal approach	AK-OTOF (Anc80L65) ~ 4.1^11^ or 8.1^11^ total vg/ear	Human	Lilly (2024)
RWM injection and stapes fenestration Sensorion delivery system	Sens-501 (AAV8) ~ 1.5 × 10¹¹ to 4.5 × 10¹¹ vg/ear	Human	Pharma (2024) and Sensorion (2024)
RWM injection 33G needle puncture 100 nL/sec with injection pump	Anc80L65-OTOF ~ 8.4 × 10¹¹–1.12 × 10¹² vg /ear Volume: 30–40 µL	Human	Otovia Therapeutics (Qi et al. 2024a; Qi et al. 2025)

The mentioned clinical studies include all five trials in children with *OTOF* variants to treat autosomal recessive nonsyndromic deafness 9 (DFNB9) involving the following companies: Refreshgene Therapeuics, Shanghai, China (ChiCTR2200063181) (Lv et al. 2024; Wang et al. 2024), Regeneron Pharmaceuticals (Decibel Therapeutics), New York, United States, (NCT05788536) (Regeneron Pharmaceuticals 2024; Valayannopoulos et al. 2025), Elli Lilly (Akouos Inc.), Indiana, United States (NCT05821959) (Lilly 2024), Otovia Therapeutics, Shanghai, China, (NCT05901480) (Qi et al. 2024a; Qi et al. 2025), Sensorion, Montpellier, France, (NCT06370351) (Pharma 2024; Sensorion 2024). Round window membrane (RWM), semicircular canal (SCC), vector genomes (vg).

## ACKNOWLEDGMENTS

The financial support by the Austrian Federal Ministry of Labour and Economy, the National Foundation for Research, Technology and Development and the Christian Doppler Research Association is gratefully acknowledged.
